# Diagnostic Performance of Rapid Antigen Testing for SARS-CoV-2: The COVid-19 AntiGen (COVAG) study

**DOI:** 10.3389/fmed.2022.774550

**Published:** 2022-03-21

**Authors:** Christoph Wertenauer, Geovana Brenner Michael, Alexander Dressel, Caroline Pfeifer, Ulrike Hauser, Eberhard Wieland, Christian Mayer, Caren Mutschmann, Martin Roskos, Hans-Jörg Wertenauer, Angela P. Moissl, Stefan Lorkowski, Winfried März

**Affiliations:** ^1^Hausärzte am Schillerplatz, Stuttgart, Germany; ^2^Medical Clinic V, Medical Faculty Mannheim, University of Heidelberg, Mannheim, Germany; ^3^Faculty of Medicine, Riga Stradins University, Riga, Latvia; ^4^SYNLAB Holding Deutschland GmbH, Augsburg, Germany; ^5^Dr. Dressel Consulting, Mannheim, Germany; ^6^SYNLAB Medical Care Center Augsburg GmbH, Augsburg, Germany; ^7^SYNLAB Medical Care Center Leinfelden-Echterdingen GmbH, Leinfelden-Echterdingen, Germany; ^8^SGS Analytics Germany GmbH, Berlin, Germany; ^9^Institute of Nutritional Sciences, Friedrich Schiller University Jena, Jena, Germany; ^10^Competence Cluster for Nutrition and Cardiovascular Health (nutriCARD) Halle-Jena-Leipzig, Jena, Germany; ^11^SYNLAB Academy, SYNLAB Holding Deutschland GmbH, Mannheim, Germany; ^12^Clinical Institute of Medical and Chemical Laboratory Diagnostics, Medical University of Graz, Graz, Austria

**Keywords:** COVID-19, SARS-CoV-2, rapid detection, antigen testing, sensitivity, diagnostic performance, variants

## Abstract

**Background:**

Rapid diagnostic testing for SARS-Cov-2 antigens is used to combat the ongoing pandemic. In this study we aimed to compare two RDTs, the SD Biosensor Q SARS-CoV-2 Rapid Antigen Test (Roche) and the Panbio COVID-19 Ag Rapid Test (Abbott), against rRT-PCR.

**Methods:**

We included 2,215 all-comers at a diagnostic center between February 1 and March 31, 2021. rRT-PCR-positive samples were examined for SARS-CoV-2 variants.

**Findings:**

Three hundred and thirty eight participants (15%) were rRT-PCR-positive for SARS-CoV-2. The sensitivities of Roche-RDT and Abbott-RDT were 60.4 and 56.8% (*P* < 0.0001) and specificities 99.7% and 99.8% (*P* = 0.076). Sensitivity inversely correlated with rRT-PCR-Ct values. The RDTs had higher sensitivities in individuals referred by treating physicians (79.5%, 78.7%) than in those referred by health departments (49.5%, 44.3%) or tested for other reasons (50%, 45.8%), in persons without any comorbidities (74.4%, 71%) compared to those with comorbidities (38.2%, 34.4%), in individuals with COVID-19 symptoms (75.2%, 74.3%) compared to those without (31.9%, 23.3%), and in the absence of SARS-CoV-2 variants (87.7%, 84%) compared to Alpha variant carriers (77.1%, 72.3%). If 10,000 symptomatic individuals are tested of which 500 are truly positive, the RDTs would generate 38 false-positive and 124 false-negative results. If 10,000 asymptomatic individuals are tested, including 50 true positives, 18 false-positives and 34 false-negatives would be generated.

**Interpretation:**

The sensitivities of the two RDTs for asymptomatic SARS-CoV-2 carriers are unsatisfactory. Their widespread use may not be effective in the ongoing SARS-CoV-2 pandemic. The virus genotype influences the sensitivity of the two RDTs. RDTs should be evaluated for different SARS-CoV-2 variants.

## Introduction

On December 31, 2019, the Municipal Health Commission of Wuhan in Hubei, China, reported a series of cases of pneumonia with unknown etiology (1). The Chinese Centre for Disease Control and Prevention (CCDC) described severe acute respiratory syndrome-related coronavirus-2 (SARS-CoV-2) as the causative agent (2–4), which then quickly spread worldwide. Severe cases of SARS-CoV-2 infection are associated with a substantial risk of prolonged critical illness and death (5).

Because the virus can be spread by asymptomatic, pre-symptomatic, and symptomatic carriers, public health experts have recommended fast and accurate testing, followed by the identification and monitoring of positive cases and subsequent self-isolation and contact tracing to contain the spread of SARS-CoV-2. Direct detection of SARS-CoV-2 is achieved by identifying viral RNA in specimens from the respiratory tract of patients utilizing nucleic acid amplification tests (NAATs) or by recognizing viral proteins through antigen-detecting rapid diagnostic tests (Ag-RDTs). The NAAT-based assays, such as real-time reverse transcription-polymerase chain reaction (rRT-PCR) have become the “gold standard” for establishing the presence of SARS-CoV-2 in patients (6). However, rRT-PCR-based testing takes several hours and is conducted in specialized laboratories, which are usually located away from sample collection sites. This may produce long turnaround times, resulting in delayed self-isolation, a risk of more contacts, and further potential transmission. Therefore, rapid antigen tests for SARS-CoV-2 detection have been made commercially available. Ag-RDTs can be conducted at the point of care and the results visualized after 15-30 min (7). There is common consensus that positive Ag-RDT results must be verified by rRT-PCR testing. Studies have indicated that the antigen tests' analytical sensitivities vary between 25 and 50% for rRT-PCR-positive samples, which may increase to more than 80% for samples with a higher viral load (8–10). We set out to comprehensively examine two of the most sensitive (11) and widely used commercial RDTs in a real-world, prospective, head-to-head study, placing specific emphasis on clinical characteristics, COVID-19-associated symptoms, and the presence of SARS-CoV-2 variant genotypes.

## Methods

### Setting and Participants

This prospective study was conducted at the Corona Test Centre Cannstatter Wasen in Stuttgart, Germany. Individuals scheduled for rRT-PCR testing of nasopharyngeal swabs were advised of the study orally and in writing. Participants had to be aged ≥ 18 years and capable of understanding the nature, significance, and implications of the study. Children and adolescents <18 years of age and patients obviously suffering from clinical conditions requiring emergency hospitalization were excluded. All participants provided written and informed consent. The study was approved by Ethics Committee II (Mannheim) of the University of Heidelberg (reference number 2020-417MF) and the German Institute for Drugs and Medical Devices.

**Figure 1:**
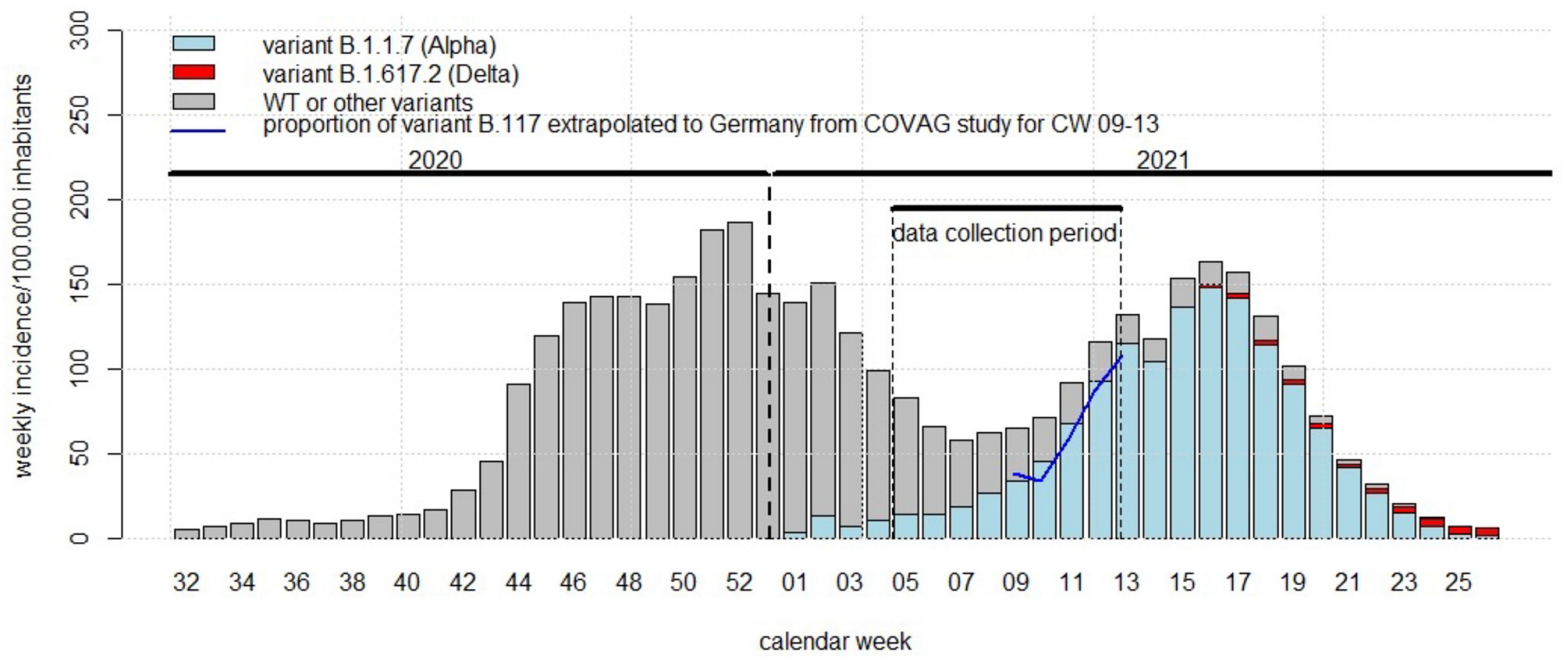
Framing of the COVAG study (February 1-March 31) into the time course of the COVID-19 pandemic in Germany. Abszissa: calendar week within 2021; ordinate: Germany-wide weekly incidence rate of SARS-Cov-2 infections per 100,000 inhabitants; bars: weekly incidence rates of infections with SARS-CoV-2 variant B.1.1.7 (alpha, blue), variant B.1.617.2 (delta, red), and wild-type or other variants (NIM-type, gray). Blue solid line: proportion of variant B.1.1.7 (alpha) in the COVAG study extrapolated to Germany.

We recorded demographic characteristics, reasons for testing, medical history, major risk factors, acute symptoms, and vital signs, including heart rate, blood pressure, body temperature, and oxygen saturation and we divided the reasons for testing into three major categories: participants referred by their primary care physicians, by the Health Department and participants who were tested for other reasons. We also framed our data collection and the emergence of variants into the course of the second and third wave of the COVID-19 pandemic in Germany (Figure 1).

Sociodemographic characteristics of the study participants were not recorded in detail upon enrollment. As a proxy, we created a sociodemographic map. For this purpose, postal codes of the study participants at baseline were merged with freely accessible data of the Robert Koch Institute (RKI, Berlin, Germany) for the German Index of Socio-Economic Deprivation (GISD) (12–14), which describes the regional socio-economic deprivation in Germany. It summarizes the extent of the socio-economic disadvantages of regions in the dimensions of education, employment, and income. The higher the GISD, the higher the deprivation. For geographic data (shape files) and regional representation, freely accessible geodata from the German Federal Office for Cartography and Geodesy (14, 15) were used. Postal codes and GISD scores were merged with the shape files with the software R version 4.1.1. and the R packages “leaflet” and “sp”; the spatial representation of a choropleth map was created to assign a GISD score to each study participant. The GISD score was represented at the district level by different colors in the choropleth map (Supplementary Figure 1).

In addition to collecting the oro- and nasopharyngeal swab for rRT-PCR testing, we collected two completely independent nasopharyngeal swab specimens to run two commercially available and widely used Ag-RDTs. The swabs were collected by medically educated personnel of the test center in changing teams with strict adherence to the instructions issued by the manufacturer. We used the Abbott Panbio^TM^ COVID-19 Ag Rapid Test (Abbott Rapid Diagnostics Jena GmbH, Jena, Germany, www.abbott.com/poct) and the Roche-SD Biosensor SARS-CoV-2 Rapid Antigen Test (identical to SD BIOSENSOR Standard Q COVID-19 Ag, www.sdbiosensor.com; Roche Diagnostics; Mannheim, Germany, www.roche.com). We chose the two tests because they were widely available and used in Germany, and because smaller studies suggested acceptable sensitivity (16–20) compared to others. While this study was ongoing, a Cochrane analysis was published which identified the SD Biosensor STANDARD Q and the Abbott Panbio test as the most sensitive amongst many others (11).

Hereafter, we refer to the tests as Abbott-RDT and Roche-RDT, respectively. We randomly assigned the participants to three sampling groups according to the time sequence of collecting the nasopharyngeal swabs (group 1: rRT-PCR, RDT-Roche, RDT-Abbott; group 2: RDT-Roche, RDT-Abbott, rRT-PCR; and group 3: RDT-Abbott, rRT-PCR, RDT-Roche).

### Analytical Procedures

Both the Abbott-RDT and the Roche-RDT were carried out by medically educated staff according to the manufacturers' instructions on-site at the Corona Test Centre Cannstatter Wasen, Stuttgart, Germany, immediately after sampling the nasopharyngeal swabs. The nasopharyngeal swabs for rRT-PCR testing were placed in 2 ml of a phosphate-buffered saline solution (ISOTON™ II Diluent, Becton Dickinson, Galway, Ireland) and delivered to the SYNLAB Medical Care Centre Leinfelden-Echterdingen. This ensured that the performers of the RDTs were unaware of the rRT-PCR-results.

SARS-CoV-2 RNA was extracted from the nasopharyngeal swab samples and purified using the PurePrep Pathogens kit and a PurePrep 96 instrument (Molgen, Veenendaal, the Netherlands) within 6 h after sampling to limit degradation. The integrity of the RNA was monitored by co-amplification of a control RNA included in the solution for the lysis of the swabs. In cases in which neither SARS-CoV-2 RNA nor the control RNA were detected, the RNA preparation was repeated. The rRT-PCR assay was performed using the RIDA®GENE SARS-CoV-2 test kit (R-Biopharm, Darmstadt, Germany) on the CFX96 Touch Real-Time PCR detection device (Bio-Rad, Feldkirchen, Germany) according to the manufacturers' instructions. This test kit targets the SARS-CoV-2 2 envelope (E) gene; samples producing a cycle threshold (Ct) ≤ 35 were considered positive by rRT-PCR.

We screened rRT-PCR-positive samples for SARS-CoV-2 variants of concern (VOC) B.1.1.7 (Alpha, United Kingdom), B.1.351 (Beta, South Africa), and P.1 (Gamma, Brazil) using VirSNiP SARS-CoV-2 Spike N501Y and VirSNiP SARS-CoV-2 Spike del H69/V70 from TIB Molbiol (Berlin, Germany) according to the supplier's instructions. Genotyping was restricted to samples with a Ct ≤ 30 in rRT-PCR (260 of 338 samples). Samples with positive results for both the N501Y substitution and H69/V70 deletion were assigned to the Alpha variant. The presence of N501Y and absence of H69/V70 was considered the Beta or Gamma variant. Samples in which both N501Y and H69/V70 were absent may have contained variants other than Alpha, Beta, or Gamma or the wild-type. For the purpose of this article, these samples were termed negative for investigated mutations (NIM).

### Statistical Analysis

**Figure 2:**
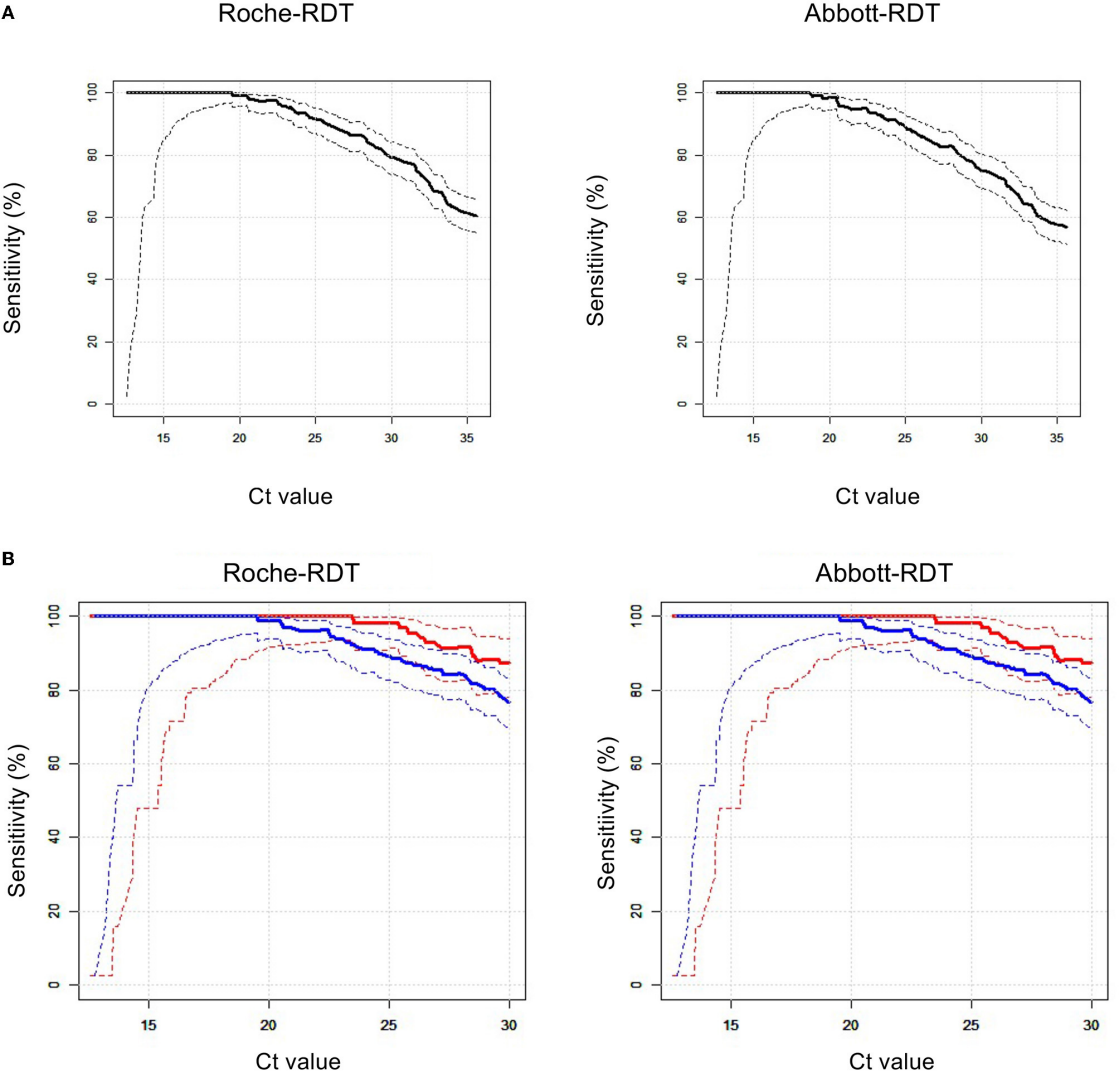
Relationships between the sensitivities of RDTs vs. rRT-PCR cycle threshold (Ct) values. The solid lines indicate sensitivities, the dotted lines represent the upper, and the lower bounds the corresponding 95% confidence intervals. **(A)** left: Roche-RDT; right: Abbott-RDT. **(B)** Sensitivities according to SARS-CoV-2 genotypes. left: Roche-RDT; right: Abbott-RDT; red: NIM genotype; blue: SARS-CoV-2 Alpha variant.

**Figure 3:**
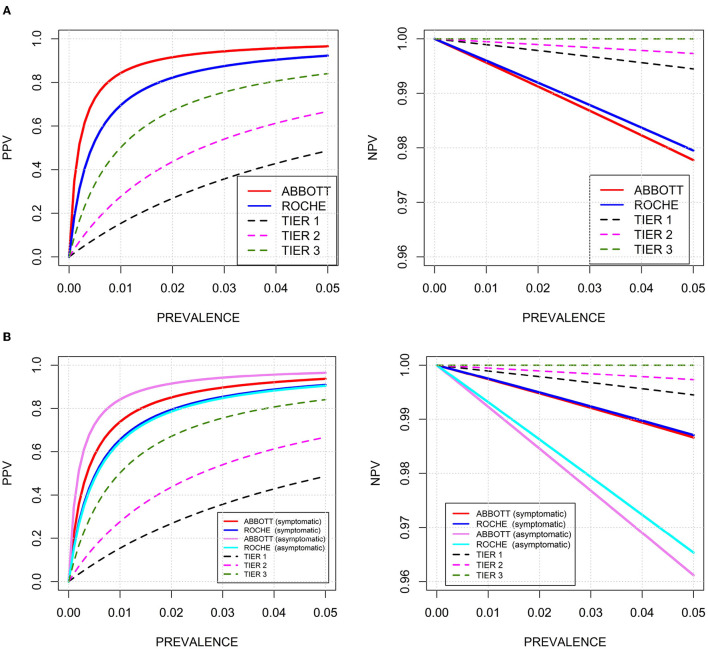
Predictive values of positive tests (PPV, left) and predictive values of negative tests (NPV, right) of two commercial RDTs for SARS-CoV-2-associated antigens in relation to disease prevalence rates up to 0.05. Black dotted line: hypothetical sensitivity and specificity of 90 and 95% (tier 1); magenta dotted line: hypothetical sensitivity and specificity of 95 and 97.5% (tier 2); green dotted line: hypothetical sensitivity and specificity of 100 and 99% (tier 3). **(A)** Red solid line: Abbott-RDT; blue solid line: Roche-RDT; **(B)** stratified according symptomatic and asymptomatic study participants. Red solid line: symptomatic participants, Abbott-RDT; blue solid line: symptomatic participants, Roche-RDT; magenta solid line: asymptomatic participants, Abbott-RDT; light blue solid line: asymptomatic participants, Roche-RDT.

Continuous data are presented as the mean, standard deviation (SD), median, and 25th and 75th percentiles. Categorical data are presented as absolute numbers and percentages. The risk of having COVID-19 according to baseline anthropometric and anamnestic characteristics was expressed in terms of crude odds ratios (ORs) and ORs adjusted for age and sex as calculated by logistic regression (Supplementary Table 1). Sensitivity, specificity, positive predictive values (PPVs), negative predictive values (NPVs), and diagnostic efficacy [(number of true positive plus true negative tests) divided by the total number of tests] of the two RDTs compared to rRT-PCR were calculated (**Table 2**). These performance indicators were compared between the Abbott-RDT and Roche-RDT. The *P*-value refers to two-sided testing of the null hypothesis, that the difference between the respective performance indicators is equal to zero and is based on 5,000 bootstrap iterations and subsequent percentile analysis. We also visualized the sensitivities of both RDTs relative to the rRT-PCR-derived Ct values (Figure 2) and the PPVs and NPVs according to hypothetical disease prevalence rates in the range of 0-0.05 (Figure 3). To compare the PPV and NPV of the RDTs with standardized criteria on performance, we also used the following hypothetical sensitivity and specificity levels (tiers 1-3) recommended by Kost (21): tier 1, 90%, 95%; tier 2, 95%, 97.5%; and tier 3, 100%, ≥99% (Figure 3).

Finally, we investigated whether the sensitivities of the two RDTs were related to the reason for testing, comorbidities, clinical symptoms, vital signs, or SARS-CoV-2 genotypes using univariate (**Table 2**) and multivariate logistic regression (**Table 3**).

The statistical tests were two-sided and *P* < 0.05 was considered significant. The analyses were carried out using R v4.0.2 (http://www.r-project.org).

## Results

### Clinical Characteristics of Participants

The study was conducted between February 1, 2021, and March 31, 2021. During this period, nearly 17,000 adult persons attended the test centre Cannstatter Wasen to receive an rRT-PCR test. A total of 2,222 persons agreed to participate in the study. Seven of them were disregarded for further evaluation because at least one of the three tests was not available. This resulted in 2,215 persons with valid data (Table 1). Adverse events from performing any of the tests were not experienced.

**Table 1:** Clinical characteristics of the 2215 participants in the COVAG study.

**Characteristics**	Numbers^*^, ^**^, ^***^
*N* =	2,215
Age, years^*^	39.9 ± 13.3, 38 (29-50)
Women^**^	1,211 (54.7)
Men^**^	1,004 (45.3)
**Reason for testing**	
Referral from physician^**^	707 (31.9)
Referral from Health Department^**^	962 (43.4)
Miscellaneous^**^	546 (24.7)
**Comorbidities**	
Any comorbidity^**^	499 (22.5)
No comorbidity^**^	1,716 (77.5)
Hypertension^**^	240 (10.8)
Dyslipoproteinaemia^**^	108 (4.9)
Diabetes mellitus^**^	49 (2.2)
COPD^**^	2 (0.8)
Ischaemic heart disease^**^	29 (1.3)
**Clinical symptoms**	
Any clinical symptoms^**^	973 (43.9)
No clinical symptoms^**^	1,242 (56.1)
Malaise^***^	632 (28.5, 65.0)
Shortness of breath^***^	181 (8.2, 18.6)
Cough^***^	459 (20.7, 47.2)
Fever^***^	149 (6.7, 15.3)
Diarrhoea^***^	154 (7.0, 15.8)
Musculoskeletal pain^***^	354 (16.0, 36.4)
Headache^***^	597 (27.0, 61.4)
Nausea^***^	129 (5.8, 13.3)
**Vaccination status**	
Not vaccinated^**^	2,016 (91)
Vaccinated^**^	198 (8.9)
Unknown^**^	1 (0)
**Vital signs (binary)**	
SysBP > 130 mmHg and/or DiasBP > 90 mmHg^*^	876 (39.5)
Other blood pressures^*^	1,339 (60.5)
Body temperature > 37°C^*^	28 (1.3)
Body temperature ≤ 37°C^*^	2,187 (98.7)
Oxygen saturation > median^*^	281 (12.7)
Oxygen saturation ≤ median^*^	1,934 (87.3)
**Vital signs (metric)**	
Systolic blood pressure, mmHg^*^	130 ± 20.2, 130 (115-140)
Diastolic blood pressure, mmHg^*^	81 ± 11.3, 80 (70-90)
Body temperature, °C^*^	36.1 ± 0.6, 36.2 (36-36.4)
Oxygen saturation, %^*^	97.2 ± 3.3, 98 (97-98)

*Data are given as n (%) or mean ± SD with median (25%, 75%)*.

* *Means ± standard deviations and medians (25th and 75th percentile)*.

** *Numbers and percentages based on the entire cohort (n = 2,215)*.

*** *Numbers and percentages based on the entire cohort (n = 2,215) and referring to patients with at least one clinical symptom*.

Figure 1 shows our data collection period within the frame of the Germany-wide weekly incidence rates and the proportion of the NIM-type, alpha (B.1.1.7) and delta (B.1.617.2) variants. During calendar weeks 9 through 13 the proportions of the B.1.1.7 in the COVAG study and in the whole of Germany completely coincided.

707 (32%) participants were referred by their primary care physicians, 962 (43%) sought testing following the advice of the Health Department and were mostly incriminated contact persons of COVID-19 patients, and 546 (25%) participants were tested for miscellaneous reasons (264 following a warning message from the German Corona App, 140 out-of-pocket payers, 82 kindergartners or teachers, and 60 for another reason, including 2 for confirmation of a positive RDT and 2 cluster students in quarantine).

When we attempted to correlate the German Index of Socio-Economic Deprivation (GISD) which is a proxy for the socioeconomic status with the risk of COVID-19, as shown in Supplementary Figure 1, 1,879 out of the 2,215 (85%) participants had a GISD between 0.55 and 0.60. Evidently, the variance of the GISD was too low to afford further analyses.

Hypertension, dyslipoproteinaemia, and diabetes mellitus were self-reported at rates of 12, 5, and 2%, respectively. Chronic obstructive lung disease and ischaemic heart disease were comparatively low in frequency. Overall, comorbidities occurred more often in men than in women (Table 1).

The most often reported clinical symptoms were malaise, headache, and musculoskeletal pain (Table 1). Symptoms were significantly more frequent in women than in men. Systolic and diastolic blood pressures were markedly and significantly higher in men than in women, but body temperature was slightly higher in women than in men (Table 1).

### Risk of rRT-PCR-Proven SARS-CoV-2 Infection According to Baseline Characteristics

Among the 2,215 participants, 338 carried SARS-CoV-2 based on rRT-PCR. Age and sex were not related to the likelihood of testing positive for SARS-CoV-2 (Supplementary Table 1). Participants referred by treating physicians and health departments were positive significantly more often than participants with miscellaneous reasons for testing [OR 0.22, 95% confidence interval (CI) 0.14-0.34, adjusted for age and sex and compared to referrals from physicians].

Persons with at least one comorbidity more frequently tested positive than those without comorbidity (OR 2.94, 95% CI 2.25-3.83, adjusted for age and sex). Among the individual comorbidities, dyslipoproteinaemia, diabetes mellitus, and ischaemic heart disease significantly increased the probability of a positive rRT-PCR test.

The presence of at least one clinical symptom at presentation resulted in a higher frequency of positive rRT-PCR (OR 2.91, 95% CI 2.28-3.71, adjusted for age and sex). In addition, each of the individual symptoms was positively and significantly related to the rate of SARS-CoV-2 detected by rRT-PCR. the objectively measured vital signs (blood pressure, body temperature, and oxygen saturation), only elevated body temperature was associated with the probability of COVID-19.

### Diagnostic Performance of RDTs

*Sensitivity*. The Roche-RDT and Abbott-RDT had overall sensitivities of 60.4 and 56.8%, respectively (*P* < 0.0001, Table 2). Figure 2A shows that the sensitivities of both RDTs were strongly related to the Ct values derived from rRT-PCR. Only at Ct values <20 did both RDTs reach a sensitivity of 100%.

**Table 2A:** Diagnostic performance of two commercial RDTs for SARS-Cov-2 antigen (part 1).

	***n* (%)**	**CT median (25th, 75th percentile) in positives**	Sensitivity (%)^*^		P^***^	Specificity (%)^**^		*P* ^***^
			**Roche-RDT**	**Abbott-RDT**		**Roche-RDT**	**Abbott-RDT**	
All probands	2,215 (100)	22.6 (18.3, 30.0)	60.4	56.8	<0.0001	99·7	99.9	0.0755
Age > median	1,099 (49.6)	24.0 (18.0, 30.5)	56.8	55.7	0.1993	99·7	99.8	0.6847
Age ≤ median	1,116 (50.4)	21.6 (18.4, 29.8)	64.2	58	<0.0001	99·8	99.9	0.4817
Women	1,211 (54.7)	23.8 (18.7, 31.1)	56.9	53.4	0.0097	99.8	99.9	0.6535
Men	1,004 (45.3)	21.6 (17.7, 29.5)	64	60.4	0.0031	99.6	99.9	0.2207
**Reason for testing**								
Referral from physician	707 (31.9)	18.7 (16.5, 24.3)	79.5	78.7	0.4971	99.5	99.8	0.2059
Referral from Health Department (mostly contact persons of infected patients)	962 (43.4)	26.0 (19.9, 31.1)	49.5	44.3	0.0003	99.9	99.9	1
Other	546 (24.7)	26.7 (19.6, 33.4)	50	45.8	0.6703	99.8	99.9	0.9964
**Comorbidities**								
Any comorbidity	499 (22.5)	28.8 (21.8, 32.5)	38.2	34.4	0.0051	99.7	99.9	0.998
No comorbidity	1,716 (77.5)	19.9 (17.5, 25.3)	74.4	71.0	0.0043	99.7	99.9	0.2263
**Clinical symptoms**								
Any clinical symptoms	973 (43.9)	19·7 (17.3, 26.0)	75.2	74.3	0.2145	99.6	99.7	0.6495
No clinical symptoms	1,242 (56.1)	29·2 (23.3, 32.6)	31.9	23.3	<0.0001	99.8	100	0.4775
**Vaccination status**								
Not vaccinated	2,016 (91)	18.3 (22.9, 30.2)	59.8	56	<0.0001	99.8	99.9	0.2255
Vaccinated	198 (8.9)	16.7 (20.4, 24.1)	73.3	73.3	0.9946	99.5	99.7	0.986
Unknown	1 (0)	^***^	^***^	^***^	^***^	^***^	^***^	^***^
**SARS-CoV-2 genotype (for Ct** **≤30)**								
N501Y and delH69/V70 (Alpha variant, B.1.1.7)	166 (7.5)	19.6 (17.0, 24.0)	77.1	72.3	0.0021			n. def.
Variants of concern not found (NIM genotypes)	81 (3.7)	19.9 (17.8, 25.0)	87.7	84	0.0635			n. def.
Other	4 (0.2)	^****^	^****^	^****^	^****^	^****^	^****^	^****^

* *Sensitivity: Proportion of people with a positive RDT related to all persons with a positive SARS-Cov-2 rRT-PCR test*.

** *Specificity: Proportion of individuals with a negative RDT to all persons with a negative e SARS-Cov-2 rRT-PCR test*.

*** *Roche-RDT vs. Abbott-RDT (test on equality, based on 5,000 bootstrap iterations)*.

**** *Not defined due to low numbers*.

**Table 2B:** Diagnostic performance of two commercial RDTs for SARS CoV-2 antigen (part 2).

	PPV (%)^*^		*P* ^****^	NPV (%)^**^		*P* ^****^	EFF (%)^***^		*P* ^****^
	**Roche-RDT**	**Abbott-RDT**		**Roche-RDT**	**Abbott-RDT**		**Roche-RDT**	**Abbott-RDT**	
All probands	97.6	99	0.0717	93.3	92.8	<0.0001	93.7	93.3	0.0037
Age > median	97.1	98	0.5867	92.4	92.2	0.2975	92.8	92.7	0.5433
Age ≤ median	98.1	99.5	0.7759	94.3	93.3	<0.0001	94.6	93.9	0.0035
Women	98	98.9	0.5437	93.2	92.7	0.0149	93.6	93.2	0.0429
Men	97.2	99	0.1865	93.4	92.8	0.0031	93.8	93.4	0.0675
**Reason for testing**									
Referral from physician	97	99	0.1377	95.9	95.7	0.7907	96	96.2	0.5533
Referral from Health Department (mostly contact persons of infected patients)	99	98.8	0.6819	88.8	87.8	0.0003	89.8	88.8	0.0003
Other	92.3	95.7	0.7045	97.7	97.6	0.3329	97.6	97.5	0.4843
**Comorbidities**									
Any comorbidity	98.0	98.9	0.5627	81.9	81	0.0025	83.6	82.7	0.0107
No comorbidity	97.5	98.7	0.2111	96.6	96.2	0.0071	96.7	96.4	0.0589
**Clinical symptoms**									
Any clinical symptoms	98.2	98.8	0.5469	93.2	92.9	0.3247	94	93.9	0.5727
No clinical symptoms	94.9	98.2	0.7659	93.4	92.7	<0.0001	93.5	92.8	0.0011
**Vaccination status**									
Not vaccinated	98	98.9	0.2143	92.9	92.3	<0.0001	93.4	92.9	0.0015
Vaccinated	91.7	95.7	0.9912	97.8	97.9	0.987	97.5	97.7	0.9888
Unknown	^*****^	^*****^	^*****^	^*****^	^*****^	^*****^	^*****^	^*****^	^*****^
**SARS-CoV-2 genotype (for Ct** **≤30)**									
N501Y and delH69/V70 (Alpha variant, B.1.1.7)	100	100	1	0	0	1	77·1	72·3	0·0021
Variants of concern not found			1			1	87·7	84	0·0635
Other	^*****^	^*****^	^*****^	^*****^	^*****^	^*****^	^*****^	^*****^	^*****^

* *PPV (predictive value of the positive tests): Proportion of true positive RDTs to all positive RDTs*.

** *PNV (predictive value of the negative tests): Proportion of true negative RDTs to all negative RDTs*.

*** *EFF (diagnostic efficiency): The ratio of correctly predicted and correctly excluded SARS-CoV-2*.

**** *Roche-RDT vs. Abbott-RDT (test on equality, based on 5000 bootstrap iterations)*.

***** *Not defined due to low numbers*.

We further examined whether the sensitivities of the two RDTs were significantly different in subgroups (Table 2). The overall difference in sensitivity between the Roche-RDT and Abbott-RDT may be due to participants below the median age (*P* < 0.0001) rather than those above the median age (*P* = 0.199, Table 2). Among participants referred by physicians, the sensitivities of both RDTs were substantially higher than in the total study population (79.5 and 78.7%, Roche-RDT and Abbott-RDT, respectively), but did not differ significantly. In contrast, they were <50% in persons referred by the Health Department and those tested for other reasons, whereby the Roche-RDT appeared to perform better than the Abbott-RDT (Table 2).

Sensitivities were markedly lower in persons with at least one comorbidity (38.2 and 34.4%, Roche-RDT and Abbott-RDT, respectively, *P* = 0.005) than in persons without comorbidities (74.4 and 71.0%, Roche-RDT and Abbott-RDT, respectively, *P* = 0.004, Table 2). This also applied to the individual comorbidities, with the one exception that the sensitivities of both the Roche-RDT and Abbott-RDT were 86% in the small number of participants reporting ischaemic heart disease (for detailed information refer to Supplementary Table 2). This unexpected finding is consistent with the Ct values being markedly higher in individuals with comorbidities (Table 2).

In persons with at least one clinical symptom, the sensitivities of both RDTs were higher (75.2 and 74.3%, Roche-RDT and Abbott-RDT, respectively, not significant) than in persons without clinical symptoms (31.9 and 23.3%, Roche-RDT and Abbott-RDT, respectively, *P* < 0.0001). The presence of any of single symptom augmented the sensitivities of both RDTs, with the exception of shortness of breath and diarrhea, which were not related to sensitivity (for detailed information refer to Supplementary Table 2). This finding is also in line with the Ct values, which were lower in cases with symptoms than in those without (Table 2).

We also analyzed whether the SARS-CoV-2 genotype affects the sensitivities of the RDTs. Only samples with Ct values ≤ 30 (*n* = 286) were included in this evaluation. The NIM SARS-CoV-2 and Alpha variant (B.1.1.7) were present in 81 and 166 samples with Ct values ≤ 30, respectively. The NIM genotype was detected at sensitivities of 87.7 and 84.0% (Roche-RDT vs. Abbott-RDT, respectively, not significant). In carriers of the Alpha variant, sensitivities were 77.1 and 72.3% (Roche-RDT vs. Abbott-RDT, respectively, *P* < 0.002). At any given Ct value, the sensitivities of both RDTs were lower for the Alpha variant than for the NIM genotype (Figure 2B).

To firmly establish independent predictors of sensitivity, we calculated ORs for having a positive RDT according to subgroups in multivariate logistic regression. Covariables were age, gender, reason for testing, presence or absence of any comorbidity, presence of absence of any clinical symptom, and the SARS-CoV-2 genotype. As expected, Ct values were associated with sensitivities of both tests. The sensitivities of the Abbott-RDT and Roche-RDT were higher in symptomatic than in asymptomatic individuals. Remarkably, the sensitivities of both tests were significantly lower for the Alpha variant than for the NIM genotypes (Table 3).

**Table 3:** Predictors of positive RDTs amongst SARS-CoV-2-rRT-PCR-positive samples in multivariate logistic regression models^*^.

		**Roche-RDT**		**Abbott-RDT**	
**Covariable**		**OR (95% CI)**	***P***	**OR (95% CI)**	***P***
Age and sex	Age, per year	0.99 (0.96, 1.02)	0.557	1.03 (0.99, 1.07)	0.098
	Men	1.0 (reference)		1.0 (reference)	
	Women	1.14 (0.46, 2.81)	0.776	0.79 (0.32, 1.94)	0.605
Ct value	Ct value on rRT-PCR, per unit	0.68 (0.6, 0.78)	**<0.0001**	0.64 (0.56, 0.74)	**<0.0001**
Reason for testing	Referral from physician	1.0 (reference)		1.0 (reference)	
	Referral from Health Department	0.53 (0.16, 1.71)	0.287	0.54 (0.17, 1.72)	0.297
	Miscellaneous reasons	1.95 (0.1, 39.67)	0.665	0.87 (0.07, 10.66)	0.914
Comorbidities	No comorbidity	1.0 (reference)		1.0 (reference)	
	Any comorbidity	0.89 (0.34, 2.33)	0.807	0.81 (0.29, 2.24)	0.681
Clinical symptoms	No clinical symptoms	1.0 (reference)		1.0 (reference)	
	At least one clinical symptom	2.22 (0.91, 5.38)	0.079	5.51 (2.18, 13.91)	**0.0003**
SARS-CoV-2 genotype	SARS-CoV-2 NIM	1.0 (reference)		1.0 (reference)	
	N501Y and delH69/V70 (alpha variant, B.1.1.7)	0.33 (0.12, 0.95	**0.040**	0.28 (0.1, 0.83)	**0.022**

* *Dependent variables: Positive result of either the Roche-RDT or the Abbott-RDT. Independent variables: age, sex, Ct value, reason for testing, comorbidities, clinical symptoms, SARS-CoV-2 genotype, all simultaneously included into two single models. This means that each OR is adjusted for the full complement of concomitant covariables. The bold values represent statistical significant values (p < 0.05)*.

*Specificity*. The rate of false-positive RDTs was low. With both RDTs, specificity exceeded 99% overall and in mostly all participant strata (Table 2, Supplementary Table 2).

*PPV, NPV, and diagnostic performance*. At a prevalence rate of 15% in the study population, the PPVs of the two RDTs were 98 and 99% and within the range of 90-100% in all subgroups examined. The NPVs of the RDTs were approximately 93%. Diagnostic efficiency also ranged between 90 and 100% (Table 2, Supplementary Table 2).

Because patients with SARS-CoV-2 infections were enriched in our study population compared to the general population, we examined the PPVs and NPVs at assumed prevalence rates up to 0.05 (Figure 3A). At this prevalence rate, our results suggest a PPV and NPV of 96.6 and 97.8% for Abbott-RDT, and 92.3 and 98.0% for the Roche-RDT, the Abbott-RDT displaying a higher PPV than the Roche-RDT and both scoring higher than the hypothetical tiers 1 through 3, reflecting increases in sensitivity in the order of Abbott-RDT < Roche-RDT < tier 1 < tier 2 < tier 3. The NPVs ranged in the order of tier 3 > tier 2 > tier 1 > Roche-RDT > Abbott-RDT.

In *symptomatic* persons, the PPVs and NPVs at the actual prevalence within the study were 98.2 and 93.2% for the Roche-RDT and 98.8 and 92.9 % for the Abbott-RDT (Table 2). In *asymptomatic* persons, the respective figures were 94.9 and 93.4% for the Roche-RDT and 98.2 and 92.7% for Abbott-RDT (Table 2). Figure 3B shows PPVs and NPVs as functions of the disease prevalence and stratified according to the *presence or absence of clinical symptoms*. As expected, PPVs were highest and NPV lowest in asymptomatic patients.

## Discussion

We completed one of the largest prospective evaluations of RDTs for SARS-CoV-2-associated antigens in a real-world environment to date. We evaluated two of the most sensitive (11) contemporary lateral-flow devices provided by Roche Diagnostics and Abbott Diagnostics. We found that the Abbott-RDT and Roche-RDT had significantly different sensitivities, but they were inversely and strongly related to the rRT-PCR-derived Ct values. In unadjusted examinations, the RDTs had higher sensitivity in individuals referred from treating physicians and health departments than in those tested for other reasons, in individuals without comorbidities compared to those with comorbidities, in individuals presenting with clinical symptoms and fever, and in carriers of the SARS-CoV-2 NIM genotype compared to carriers of the Alpha variant. Of note, the significant negative association between positivity of RDT results and presence of the alpha variant remains statistically significant when we considered age, sex, Ct-value, reason for testing, presence of comorbidities and presence of clinical symptoms in the same logistic regression model. This implies that the RDTs are less sensitive toward the alpha variant even adjusting for other conditions affecting sensitivity.

### Prevalence of SARS-CoV-2 Infections in the Study Cohort

Among the attendees of our corona test center, the rate of individuals testing positive for SARS-CoV-2 infection by rRT-PCR was 15%, which markedly exceeds the prevalence rate in the German population during the study (~0.3% estimated on the basis of 7-day incidence rates during the study period). The probability of testing positive by rRT-PCR was not related to age and sex. This is in contrast to the expected over-representation of older people and may reflect pre-selection for persons having an evident clinical indication for testing. The probability of testing positive by rRT-PCR was strongly linked to individual reasons for testing, to the presence of comorbidities, clinical complaints, and elevated body temperature. For example, the persons referred by physicians due to suspected COVID-19 or those coming from the Health Department were more often positive than the group with miscellaneous reasons (kindergartners/teachers, out-of-pocket payers). Yet, in the latter group (prevalence rate 0.044), the proportion of SARS-CoV-2 carriers was still 10-fold higher than in the general population (assumed prevalence 0.003).

### Sensitivity of the RDTs

Expectedly, both RDTs had higher sensitivities in subgroups with high viral loads (referral by physicians and health departments, clinical symptoms). However, both sensitivities and viral loads were low in patients presenting with comorbidities. This is unexpected and may reflect a referral bias in the sense that the indication for testing is more frequent and earlier in patients at high risk for severe COVID-19.

The relationship between the RDTs' analytical sensitivity and viral load is in line with reports of sensitivities between 24.3 and 50% for RT-PCR-positive samples, which increased up to 81.8 and 100% for samples with high viral loads (>6 log_10_ RNA copies/ml) (8–10). In specimens from the upper respiratory tract, SARS-CoV-2 RNA peaks with the beginning of symptoms around day 4, decreases steadily during the first 10 days after illness onset, and can be detected up to 20 days after the onset of symptoms (22–25). However, viral loads are low during the earlier stage of infection and in the second week after the onset of symptoms (26, 27). Thus, the time window for the detection of SARS-CoV-2 by RDTs appears to be narrower than with rRT-PCR and may be confined to the acute phase of infection. Consistently, we found a lower sensitivity of RDTs in asymptomatic individuals, suggesting limited usefulness of RDTs for screening such individuals, even if it is repeated on a regular basis.

We want to emphasize that the RDTs were carried out by medically educated personnel with strict adherence to the instructions issued by the manufacturers, perhaps explaining the low rate of false-positive results. Yet, RDTs have been widely recommended for self-testing or for testing by lay persons. Indeed, when Ag-RDT self-testing results were evaluated in a comparative study among *symptomatic* outpatients, self-testing (including self-read-out) yielded a sensitivity of 82.5% compared to professional nasopharyngeal sampling and testing (28). The same study noted variations in the sensitivity of Ag-RDT self-testing depending on the viral load of the sample. High viral loads ≥ 7.0 log10 SARS-CoV-2 RNA copies/ml led to a sensitivity of 96.6%, whereas low viral loads <7.0 log10 SARS-CoV-2 RNA copies/ml had decreased sensitivity (45.6% for Ag-RDT self-use and 54.5% for Ag-RDT professional use) (28). Thus, it appears that, at low viral loads, as encountered in *asymptomatic* persons, self-administered RDT testing may be even less effective in reality than it was in the current study.

### Sensitivity for the SARS-CoV-2 Alpha Variant

During the conduct of our study, the SARS-CoV-2 Alpha variant became the prevailing genotype in Southern Germany. Consequently, the study included 81 carriers of SARS-CoV-2 NIM, 166 carriers of the Alpha variant, and 4 patients with other viral genotypes (251 of 338 samples with Ct values ≤ 30). Remarkably, the sensitivities of both RDTs were lower in Alpha variant carriers, and this finding was robust against adjustments for the viral load expressed in terms of Ct values (Table 3). The Alpha variant may be differentiated from the wild-type by two key mutations in the Spike protein: the N501Y substitution within the receptor-binding domain and the H69/V70 deletion. It may be ~80% more transmissible than the wild-type (29) due to conformational changes increasing the Spike protein's affinity for the angiotensin-converting enzyme 2 (ACE2) receptor (30–32). SARS-CoV-2 variants may also confer decreased binding of therapeutic antibodies and protection by vaccination, whereby the Alpha variant may display the smallest variation in antigenicity compared to other circulating variants. This raises the attractive possibility that the lower reactivity of the current RDTs for the Alpha variant was related to structural alterations in the epitope(s) recognized by the detecting antibodies. However, the antibodies incorporated in both RDTs recognize the nucleocapsid protein (N-protein) (33). In a laboratory-based investigation (virus suspended in cell culture medium and saliva), the performance of the Roche-RDT and Abbott-RDT was not affected by variants. This is in contrast to the current results, which were collected in a real-world setting. A potential speculative explanation for the discrepancies may be that both the Spike protein and the N-protein tightly interact, and that conformational changes in the Spike protein may affect the three-dimensional structure and accessibility for antibodies against the N-protein (34). Finally, we cannot rule out that the structural changes in other variants may be even greater than in the Alpha variant (35), so that their effect on sensitivity may be even stronger. Thus, any validation of RDTs for SARS-CoV-2 would also have to be extended to known and future variants rather than limited to the wild-type SARS-CoV-2.

### Implications for Screening

A recent meta-analysis issued by the Cochrane Collaboration reviewed 48 studies including 58 commercial RDTs. It reported sensitivities between 34.1% (95% CI 29.7-38.8%) and 88.1% (95% CI 84.2-91.1%) for RDTs in *symptomatic* persons, whereas in *asymptomatic* persons, the sensitivities varied between 28.6%(8.4-58.1%) and 69.2% (38.6-90.9%) (11).

The Cochrane analysis identified three studies of the Abbott-RDT with 1,094 *symptomatic* participants, including 252 SARS-CoV-2 cases, and one study with 474 *asymptomatic* persons and 47 cases. It also identified three studies with 1,948 *symptomatic* participants and 336 cases and one study with 127 *asymptomatic* persons and 13 cases for the Roche-RDT (listed as Biosensor Standard Q). The number of *asymptomatic* persons in the current single study thus the number in the study in the Cochrane meta-analysis (11).

For the Abbott-RDT, the Cochrane analysis reported sensitivities of 75.1% (57.3-87.1%) and 48.9% (35.1-62.9%), and specificities of 99.5% (98.7-99.8%) and 98.1% (96.3-99.1%) in *symptomatic* and *asymptomatic* persons, respectively. The sensitivity of the Roche-RDT was reported to be 88.1% (84.2-91.1%) and 69.2% (38.6-90.9%) in *symptomatic* and *asymptomatic* patients, respectively, with specificities similar to the Abbott-RDT. The current findings therefore almost exactly coincide with the Cochrane analysis and significantly extends the available evidence.

Analogous with the viewpoint of the Cochrane analysis (11), at a prevalence rate of 0.05, sensitivities of 60.4 and 56.8% (symptomatic: 75.23 and 74.32%, asymptomatic: 31.9 and 23.28%) and specificities of 99.7 and 99.9% (symptomatic: 99.6 and 99.73%, asymptomatic: 99.82 and 99.96%) with the Roche-RDT and Abbott-RDT, respectively, in *symptomatic* patients with SARS-CoV-2 infections, our data would translate as follows.

If 10,000 patients with expected 500 (0.05) true positives were examined, 414 and 397 persons would have tested positive, of which 38 and 25 would have been false-positives and 124 and 128 persons with negative test results would be falsely negative for Roche-RDT and Abbott-RDT, respectively (Table 4). Assuming a prevalence rate of 0.005 with expected 50 true positives in 10,000 *asymptomatic* patients, there would be 34 and 16 persons testing positive, of which 18 and 4 would have been false-positives for Roche-RDT and Abbott-RDT, respectively, and 34 and 38 persons with a negative test would be falsely negative (Table 4). This is crucially important, as RDTs have specifically been recommended for screening asymptomatic persons. Furthermore, as shown in Table 4, these numbers would be affected if the SARS-CoV-2 Alpha variant is predominant.

**Table 4A:** Number of false-positive and false-negative results in a hypothetical cohort of 10,000 people tested with the Roche-RDT.

**Hypothetical cohort (assumed number = 10,000)**	**Prevalence rate**	**Expected number of true positives**	**Sensitivity (%)**	**Specificity (%)**	Number of false positives (95% CI)^*^	Number of false negatives (95% CI)^*^
Symptomatic persons	0.05	500	75.23	99.60	38 (26, 51)	124 (103, 147)
	0.10	1,000	75.23	99.60	36 (25, 48)	248 (218, 278)
	0.20	2,000	75.23	99.60	32 (22, 44)	495 (454, 537)
Symptomatic persons: SARS-CoV-2 NIM	0.05	500	79.30	99.60	38 (26, 51)	104 (84, 123)
	0.10	1,000	79.30	99.60	36 (25, 48)	207 (180, 235)
	0.20	2,000	79.30	99.60	32 (21, 43)	414 (376, 454)
Symptomatic persons: SARS-CoV-2 Alpha variant	0.05	500	71.76	99.60	38 (26, 50)	141 (119, 165)
	0.10	1,000	71.76	99.69	36 (25, 48)	282 (251, 315)
	0.20	2,000	71.76	99.60	32 (21, 44)	564 (519, 609)
Asymptomatic persons	0.005	50	31.90	99.82	18 (10, 26)	34 (23, 46)
	0.01	100	31.90	99.82	18 (10, 26)	68 (52, 85)
	0.02	200	31.90	99.82	17 (10, 26)	136 (115, 159)
Asymptomatic persons: SARS-CoV-2 NIM	0.005	50	32.65	99.82	18 (10, 26)	34 (23, 45)
	0.01	100	32.65	99.82	18 (10, 26)	67 (52, 84)
	0.02	200	32.65	99.82	17 (10, 26)	135 (113, 157)
Asymptomatic persons: SARS-CoV-2 Alpha variant	0.005	50	30.75	99.82	18 (10, 27)	35 (24, 47)
	0.01	100	30.75	99.82	18 (10, 26)	69 (53, 85)
	0.02	200	30.75	99.82	17 (10, 26)	139 (116, 162)

* *mean (2.5%,97.5%)*.

**Table 4B:** Number of false-positive and false-negative results in a hypothetical cohort of 10 000 people tested with the Abbott –RDT.

**Hypothetical cohort (assumed number = 10,000)**	**Prevalence rate**	**Expected number of true positives**	**Sensitivity (%)**	**Specificity (%)**	Number of false positives (95% CI)^*^	Number of false negatives (95% CI)^*^
Symptomatic persons	0.05	500	74.32	99.73	25 (16, 36)	128 (107, 151)
	0.10	1,000	74.32	99.73	24 (15, 34)	257 (227, 287)
	0.20	2,000	74.32	99.73	21 (13, 31)	513 (472, 556)
Symptomatic persons: SARS-CoV-2 NIM	0.05	500	78.10	99.73	25 (16, 36)	110 (90, 130)
	0.10	1,000	78.10	99.73	24 (15, 34)	219 (191, 248)
	0.20	2,000	78.10	99.73	21 (13, 31)	438 (398, 479)
Symptomatic persons: SARS-CoV-2 Alpha variant	0.05	500	71.12	99.73	25 (16, 36)	144 (121, 169)
	0.10	1,000	71.12	99.73	24 (15, 34)	289 (256, 322)
	0.20	2,000	71.12	99.73	21 (13, 31)	577 (532, 622)
Asymptomatic persons	0.005	50	23.28	99.96	4 (1, 9)	38 (27, 51)
	0.01	100	23.28	99.96	4 (1, 9)	77 (60, 94)
	0.02	200	23.28	99.96	4 (1, 9)	153 (130, 178)
Asymptomatic persons: SARS-CoV-2 NIM	0.005	50	24.39	99.96	4 (1, 9)	38 (27, 50)
	0.01	100	24.39	99.96	4 (1, 9)	76 (59, 93)
	0.02	200	24.39	99.96	4 (1, 9)	152 (128, 176)
Asymptomatic persons: SARS-CoV-2 Alpha variant	0.005	50	21.58	99.96	4 (1, 9)	39 (28, 52)
	0.01	100	21.58	99.96	4 (1, 9)	78 (61, 96)
	0.02	200	21.58	99.96	4 (1, 9)	157 (133, 183)

* *mean (2.5%,97.5%)*.

### Limitations

We applied rRT-PCR as the reference method to detect SARS-CoV-2 infection. Despite being considered the gold standard, this technique has the limitation that the detection of SARS-CoV-2 RNA in patient samples does not indicate the presence or shedding of viable virus with replicative capacity or whether the tested individual is contagious at the time of the test (22, 36). Although the presence of viral RNA proven by rRT-PCR does not automatically equate to infectiousness, a significant correlation between the Ct value (reflecting viral load) and subsequent virus cultivation has been observed. Samples with Ct values between 13 and 17 have a culture positivity rate of 100%, which declines gradually to 12% when Ct values of 33 are analyzed. No viral growth occurs at Ct values ≥ 34, suggesting that patients with these values do not excrete infectious viral particles (27). Therefore, if infectivity rather than a positive rRT-PCR test were considered the reference for RDTs, our results would stand more in favor of RDT testing. However, a direct conversion of Ct values or a positive RDT to contagiousness has not yet been established. Ct values can hardly be compared across studies, and the correlation between viral load and the risk of transmission from a positive case is still not entirely clear (23, 37), with a variety of circumstances, such as the individual's behavior, the type and duration of contact, the environment, and the implementation of transmission-reducing measures (e.g., filter masks) affect infectiveness (37, 38).

The sensitivity of RDTs may relate to the time elapsed since infection. It is a limitation of the current study that the time point of infection in rRT-PCR-positive samples was not available. However, we consider the Ct values from rRT-PCR as a good proxy for the changes in viral load during the course of the study.

Finally, we demonstrated that the SARS-CoV-2 Alpha variant markedly diminishes the sensitivity of both RDTs. We cannot explain this finding. Other variants were not encountered in sufficient numbers, and we were not able to infer the sensitivities of the RDTs for other variants.

### Directions for Future Research

This evaluation of two of the most sensitive RDTs currently available for SARS-CoV-2 suggests that screening asymptomatic persons with this approach may fail to identify a substantial proportion of viral carriers. Thus, further methodical refinements are needed, such as attempts to determine the viral load at least semi-quantitatively. Alternatively, rapid, on-site, direct detection of SARS-CoV-2 RNA RDTs could be pursued. The lower sensitivity of the RDTs for the Alpha variant indicates that their performance may be substantially influenced by the virus genotype, and strategies need to be developed to ensure that any of the circulating variants are captured. Finally, further head-to-head research is needed into how current screening strategies, RDT or laboratory-based, directly translate into controlling virus transmission and spread in the population. This will show whether the obvious practical advantage of RDTs is offset by their limitations.

## Data Availability Statement

Data will be made available to researchers upon justified request and formal agreement to make sure that rules of good scientific practice are obeyed and that credit is given to the people who have been in charge of the design and the organization of the study. Interested researchers are invited to address their request or proposal to WM (winfried.maerz@synlab.com). The authors confirm that they accessed and validated these data and that all other researchers can access the data in the same manner the authors did.

## Ethics Statement

The studies involving human participants were reviewed and approved by Ethics Committee II (Mannheim) of the University of Heidelberg (reference number 2020-417MF). The patients/participants provided their written informed consent to participate in this study.

## Author Contributions

WM, CMa, and CMu designed the study. CW, GB, UH, and H-JW collected the data. AD performed the statistical analysis. EW and GB surveyed the laboratory analyses. AM and SL examined the sociodemographic characteristics of study participants. CW and WM wrote the manuscript. All authors validated, reviewed, and edited the manuscript.

## Funding

The costs of the study were defrayed by SYNLAB Holding Deutschland GmbH. The management had no role in writing of the report or the decision to submit for publication. There was no financial support to SYNLAB Holding Deutschland GmbH from the manufacturers of the assays used in this evaluation and there has been no other financial support for this work that could have influenced its outcome.

## Conflict of Interest

CW, GB, CP, UH, EW, CMa, MR, and WM were employed by SYNLAB Holding Germany GmbH or its regional subsidiaries. AD is the owner of Company Dr. Dressel Consulting. CMu was employed by company SGS Analytics Germany GmbH. The remaining authors declare that the research was conducted in the absence of any commercial or financial relationships that could be construed as a potential conflict of interest.

## Publisher's Note

All claims expressed in this article are solely those of the authors and do not necessarily represent those of their affiliated organizations, or those of the publisher, the editors and the reviewers. Any product that may be evaluated in this article, or claim that may be made by its manufacturer, is not guaranteed or endorsed by the publisher.

## Abbreviations

NIM: negative for investigated mutations. Samples in which both N501Y and H69/V70 were absent may have contained variants other than Alpha, Beta, or Gamma or the wild-type. For the purpose of this article these samples were termed negative for investigated mutations (NIM).
