# Supplementary material for: Diagnostic Performance of Rapid Antigen Testing for SARS-CoV-2: The COVid-19 AntiGen (COVAG) study
Source: Front Med (Lausanne). 2022 Mar 21;9:774550. doi: 10.3389/fmed.2022.774550 (PMC8979030; doi:10.3389/fmed.2022.774550)

**Supplementary Material**

**Frontiers of Medicine – MS Number 774550**

**Diagnostic Performance of Rapid Antigen Testing for SARS-CoV-2:**

**The COVid-19 AntiGen (COVAG) study**

Christoph Wertenauer^1,2^*^,3,4^, Geovana Brenner Michael^4^, Alexander Dressel^5^, Caroline Pfeifer^4^, Ulrike Hauser^6^, Eberhard Wieland^7^, Christian Mayer^4^, Caren Mutschmann^8^, Martin Roskos^4^, Hans-Jörg Wertenauer^1^, Angela P. Moissl^9,10,2^, Stefan Lorkowski^9,10^, Winfried März^2,11,12^

1. Hausärzte am Schillerplatz, Stuttgart, Germany
2. Medical Clinic V, Medical Faculty Mannheim, University of Heidelberg, Mannheim, Germany
3. Riga Stradins University, Riga, Latvia
4. Synlab Holding Deutschland GmbH, Augsburg, Germany
5. Dr. Dressel Consulting, Mannheim, Germany
6. SynlaB Medical Care Center Augsburg GmbH, Augsburg, Germany
7. SynlaB Medical Care Center Leinfelden-Echterdingen GmbH, Leinfelden-Echterdingen, Germany
8. SGS Analytics Germany GmbH, Berlin, Germany
9. Institute of Nutritional sciences, Friedrich Schiller University Jena, Jena, Germany
10. Competence Cluster for Nutrition and Cardiovascular Health (nutriCARD) Halle-Jena-Leipzig, Jena, Germany
11. SYNLAB Academy, SYNLAB Holding Deutschland GmbH, Mannheim, Germany
12. Clinical Institute of Medical and Chemical Laboratory Diagnostics, Medical University of Graz, Graz, Austria

*** Correspondence:**Christoph Wertenauer
christoph.wertenauer@uni-heidelberg.de

| **Supplementary Table 1. Risk for rRT-PCR-proven SARS2-Cov-2 infection according to clinical characteristics in 2215 participants of the COVAG study.** | | | | |  |  |  |
| --- | --- | --- | --- | --- | --- | --- | --- |
|  |  |  |  |  |  | |  |
|  | **N** | **PCR Positive, N (%)** | **OR (95% CI)** | **OR (95% CI)** | |  | |
|  |  |  | **Crude** | **Adjusted for age and sex** | |  | |
| Age (per year) * |  |  | 1 (0·99, 1·01) |  | |  | |
| Male sex (reference: female) | 1004 | 164 (16·3) | 1·16 (0·92, 1·47) |  | |  | |
| **REASON FOR TESTING** |  |  |  |  | |  | |
| Referral from physician | 707 | 122 (17·3) | 1·0 (reference) | 1·0 (reference) | |  | |
| Referral from Health Department (mostly contact persons of infected patients) | 962 | 192 (20) | 1·2 (0·93, 1·54) | 1·2 (0·93, 1·54) | |  | |
| Other | 546 | 24 (4·4) | 0·22 (0·14, 0·35) | 0·22 (0·14, 0·34) | |  | |
| **COMORBIDITIES** |  |  |  |  | |  | |
| Any comorbidity | 499 | 131 (26·3) | 2·6 (2·03, 3·32) | 2·94 (2·25, 3·83) | |  | |
| No comorbidity | 1716 | 207 (12·1) | 1·0 (reference) | 1·0 (reference) | |  | |
| Hypertension | 240 | 37 (15·4) | 1·33 (0·91, 1·94) | 1·46 (0·97, 2·2) | |  | |
| Dyslipoproteinaemia | 108 | 18 (16·7) | 1·46 (0·86, 2·47) | 1·76 (1·01, 3·05) | |  | |
| Diabetes mellitus | 49 | 10 (20·4) | 1·87 (0·92, 3·8) | 2·24 (1·08, 4·63) | |  | |
| COPD | 17 | 2 (11·8) | 0·97 (0·22, 4·28) | 1·19 (0·27, 5·27) | |  | |
| Ischaemic heart disease | 29 | 7 (24·1) | 2·32 (0·98, 5·5) | 2·87 (1·19, 6·91) | |  | |
| Previous COVID-19 | 182 | 99 (54·4) | 8·7 (6·28, 12·04) | 8·61 (6·21, 11·93) | |  | |
| **CLINICAL SYMPTOMS** |  |  |  |  | |  | |
| Any clinical symptoms | 973 | 222 (22·8) | 2·87 (2·25, 3·66) | 2·91 (2·28, 3·71) | |  | |
| No clinical symptoms | 1242 | 116 (9·3) | 1·0 (reference) | 1·0 (reference) | |  | |
| Malaise | 632 | 155 (24·5) | 3·15 (2·42, 4·1) | 3·2 (2·46, 4·17) | |  | |
| Shortness of breath | 181 | 48 (26·5) | 3·5 (2·39, 5·13) | 3·6 (2·45, 5·28) | |  | |
| Cough | 459 | 133 (29) | 3·96 (3, 5·23) | 4·01 (3·03, 5·3) | |  | |
| Fever | 149 | 58 (38·9) | 6·19 (4·23, 9·05) | 6·17 (4·22, 9·04) | |  | |
| Diarrhoea | 154 | 32 (20·8) | 2·55 (1·65, 3·93) | 2·58 (1·67, 3·98) | |  | |
| Musculoskeletal pain | 354 | 114 (32·2) | 4·61 (3·44, 6·18) | 4·67 (3·48, 6·26) | |  | |
| Headache | 597 | 128 (21·4) | 2·65 (2·02, 3·48) | 2·7 (2·05, 3·56) | |  | |
| Nausea | 129 | 25 (19·4) | 2·33 (1·45, 3·76) | 2·42 (1·5, 3·91) | |  | |
| **VACCINATION STATUS** |  |  |  |  | |  | |
| Not vaccinated | 2016 | 323 (16) | 2·33 (1·36, 3·99) | 2·28 (1·32, 3·92) | |  | |
| Vaccinated | 198 | 15 (7·6) | 1·0 (reference) | 1·0 (reference) | |  | |
| Unknown | 1 | 0 (0) | ** | ** | |  | |
| **VITAL SIGNS** |  |  |  |  | |  | |
| SysBP>130 mmHg and/or SysBP>90 mmHg | 876 | 125 (14·3) | 0·88 (0·69, 1·12) | 0·84 (0·65, 1·09) | |  | |
| Body temperature > 37°C | 28 | 12 (42·9) | 4·28 (2·01, 9·13) | 4·4 (2·05, 9·41) | |  | |
| Oxygen saturation > median | 281 | 38 (13·5) | 0·85 (0·59, 1·22) | 0·87 (0·6, 1·26) | |  | |
|  |  |  |  |  | |  | |
| * For metric variables, the estimated increase in log-odds for rRT-PCR-proven SARS2-Cov-2 infection per one-unit increase. | | | | |  | |  |
| ** Not calculated due to low number | | | | |  | |  |

| \| **Supplementary Table 2. Diagnostic performance of two commercial RDTs for SARS-Cov-2 antigen (part 1)** \| \| \| \| \| \| \| \| \| \| --- \| --- \| --- \| --- \| --- \| --- \| --- \| --- \| --- \| \| \|  \| **n (%)** \| **CT median (25th, 75th percentile)** \| **Sensitivity (%)** \| \| **P*** \| **Specificity (%)** \| \| **P*** \| \|  \|  \| **in positives** \| **Roche-RDT** \| **Abbott-RDT** \|  \| **Roche-RDT** \| **Abbott-RDT** \|  \| \| All probands \| 2215 (100) \| 22·6 (18·3, 30) \| 60·4 \| 56·8 \| <0·0001 \| 99·7 \| 99·9 \| 0·0755 \| \| Age > median \| 1099 (49·6) \| 24 (18, 30·5) \| 56·8 \| 55·7 \| 0·1993 \| 99·7 \| 99·8 \| 0·6847 \| \| Age ≤ median \| 1116 (50·4) \| 21·6 (18·4, 29·8) \| 64·2 \| 58 \| <0·0001 \| 99·8 \| 99·9 \| 0·4817 \| \| Women \| 1211 (54·7) \| 23·8 (18·7, 31·1) \| 56·9 \| 53·4 \| 0·0097 \| 99·8 \| 99·9 \| 0·6535 \| \| Men \| 1004 (45·3) \| 21·6 (17·7, 29·5) \| 64 \| 60·4 \| 0·0031 \| 99·6 \| 99·9 \| 0·2207 \| \| **Reason for testing** \|  \|  \|  \|  \|  \|  \|  \|  \| \| Referral from physician \| 707 (31·9) \| 18·7 (16·5, 24·3) \| 79·5 \| 78·7 \| 0·4971 \| 99·5 \| 99·8 \| 0·2059 \| \| Referral from Health Department (mostly contact persons of infected patients) \| 962 (43·4) \| 26 (19·9, 31·1) \| 49·5 \| 44·3 \| 0·0003 \| 99·9 \| 99·9 \| 1 \| \| Other \| 546 (24·7) \| 26·7 (19·6, 33·4) \| 50 \| 45·8 \| 0·6703 \| 99·8 \| 99·9 \| 0·9964 \| \| **Comorbidities** \|  \|  \|  \|  \|  \|  \|  \|  \| \| Any comorbidity \| 499 (22·5) \| 28·8 (21·8, 32·5) \| 38·2 \| 34·4 \| 0·0051 \| 99·7 \| 99·9 \| 0·998 \| \| No comorbidity \| 1716 (77·5) \| 19·9 (17·5, 25·3) \| 74·4 \| 71 \| 0·0043 \| 99·7 \| 99·9 \| 0·2263 \| \| Hypertension \| 240 (10·8) \| 27·5 (20·3, 32·3) \| 51·4 \| 51·4 \| 1 \| 99·8 \| 99·8 \| 1 \| \| Dyslipoproteinaemia \| 108 (4·9) \| 27·8 (19·4, 32·1) \| 55·6 \| 55·6 \| 1 \| 98·9 \| 99·4 \| 0·9748 \| \| Diabetes mellitus \| 49 (2·2) \| 22·2 (17·3, 29·5) \| 50 \| 50 \| 1 \| 97·4 \| 98·7 \| 0·9908 \| \| COPD \| 2 (0·8) \| *** \| *** \| *** \| *** \| *** \| *** \| *** \| \| Ischaemic heart disease \| 29 (1·3) \| 19·8 (16·8, 22·9) \| 85·7 \| 85·7 \| 1 \| 95·5 \| 97·7 \| 0·9994 \| \| Previous COVID-19 \| 182 (8·2) \| 30 (25·4, 32·6) \| 28·3 \| 23·2 \| 0·0071 \| 99·4 \| 99·4 \| 1 \| \| **Clinical symptoms** \|  \|  \|  \|  \|  \|  \|  \|  \| \| Any clinical symptoms \| 973 (43·9) \| 19·7 (17·3, 26) \| 75·2 \| 74·3 \| 0·2145 \| 99·6 \| 99·7 \| 0·6495 \| \| No clinical symptoms \| 1242 (56·1) \| 29·2 (23·3, 32·6) \| 31·9 \| 23·3 \| <0·0001 \| 99·8 \| 100 \| 0·4775 \| \| Malaise \| 632 (28·5, 65·0) \| 19·3 (16·9, 24·6) \| 81·9 \| 81·3 \| 0·4981 \| 99·6 \| 99·8 \| 0·6667 \| \| Shortness of breath \| 181 (8·2, 18·6) \| 21·8 (18·1, 32·4) \| 58·3 \| 58·3 \| 1 \| 99·6 \| 99·6 \| 1 \| \| Cough \| 459 (20·7, 47·2) \| 19·9 (17·3, 25·4) \| 75·9 \| 75·2 \| 0·4951 \| 99·8 \| 99·8 \| 1 \| \| Fever \| 149 (6·7, 15·3) \| 18·4 (15·8, 21·3) \| 89·7 \| 87·9 \| 0·6775 \| 99·5 \| 99·5 \| 1 \| \| Diarrhoea \| 154 (7·0, 15·8) \| 21·4 (18·2, 29·3) \| 65·6 \| 65·6 \| 1 \| 99·2 \| 99·6 \| 0·9876 \| \| Musculoskeletal pain \| 354 (16·0, 36·4) \| 18·6 (16·3, 22·6) \| 82·5 \| 82·5 \| 0·9934 \| 99·6 \| 99·8 \| 0·9978 \| \| Headache \| 597 (27·0, 61·4) \| 19 (16·9, 24·2) \| 81·2 \| 80·5 \| 0·5083 \| 99·6 \| 99·8 \| 0·6563 \| \| Nausea \| 129 (5·8, 13·3) \| 18·6 (16·8, 24·5) \| 76 \| 72 \| 0·6915 \| 99 \| 99·5 \| 0·999 \| \| **Vaccination status** \|  \|  \|  \|  \|  \|  \|  \|  \| \| Not vaccinated \| 2016 (91) \| 18·3 (22·9, 30·2) \| 59·8 \| 56 \| <0·0001 \| 99·8 \| 99·9 \| 0·2255 \| \| Vaccinated \| 198 (8·9) \| 16·7 (20·4, 24·1) \| 73·3 \| 73·3 \| 0·9946 \| 99·5 \| 99·7 \| 0·986 \| \| Unknown \| 1 (0) \| *** \| *** \| *** \| *** \| *** \| *** \| *** \| \| **Vital signs** \|  \|  \|  \|  \|  \|  \|  \|  \| \| SysBP>130 mmHg and/or SysBP>90 mmHg \| 876 (39·5) \| 24·1 (17·7, 31·6) \| 54·4 \| 54·4 \| 1 \| 99·7 \| 99·9 \| 0·4899 \| \| Other blood pressures \| 1339 (60·5) \| 22·5 (18·4, 29·4) \| 63·8 \| 58·2 \| <0·0001 \| 99·7 \| 99·8 \| 0·6563 \| \| Body temperature > 37°C \| 28 (1·3) \| 18 (16·4, 22·2) \| 91·7 \| 91·7 \| 1 \| 96·9 \| 96·9 \| 1 \| \| Body temperature ≤ 37°C \| 2187 (98·7) \| 23·1 (18·4, 30·2) \| 59·2 \| 55·5 \| <0·0001 \| 99·7 \| 99·9 \| 0·0759 \| \| Oxygen saturation > median \| 281 (12·7) \| 23 (19·8, 29·8) \| 57·9 \| 57·9 \| 1 \| 99·8 \| 99·8 \| 1 \| \| Oxygen saturation ≤ median \| 1934 (87·3) \| 22·6 (18·2, 30) \| 60·7 \| 56·7 \| 0·0003 \| 99·7 \| 99·9 \| 0·0627 \| \| **SARS-CoV-2 genotype (for Ct≤30)** \|  \|  \|  \|  \|  \|  \|  \|  \| \| N501Y and delH69/V70 (Alpha variant, B.1.1.7) \| 166 (7·5) \| 19·6 (17, 24) \| 77·1 \| 72·3 \| 0·0021 \|  \|  \| not defined \| \| Variants of concern not found \| 81 (3·7) \| 19·9 (17·8, 25) \| 87·7 \| 84 \| 0·0635 \|  \|  \| not defined \| \| Other \| 4 (0·2) \| *** \| *** \| *** \| *** \| *** \| *** \| *** \| \|  \|  \|  \|  \|  \|  \|  \|  \|  \| \| **** Roche versus Abbott (test on equality, based on 5000 bootstrap iterations). \| \| \| \| \|  \|  \|  \|  \| \| **** No entry (division by zero, etc.). \| \| \| \| \|  \|  \|  \|  \| \| **** Not defined due to low number. \| \| \| \| \|  \|  \|  \|  \| \| **** Too low number of patients with negative/positive RDT. \| \| \| \| \|  \|  \|  \|  \| \| Sensitivity: Proportion of people with a positive RDT related to all persons with a positive SARS-Cov-2 rRT-PCR test. \| \| \| \| \|  \|  \|  \|  \| \| Specificity: Proportion of individuals with a negative RDT to all persons with a negative e SARS-Cov-2 rRT-PCR test. \| \| \| \| \|  \|  \|  \|  \|  \| **Supplemetary Table 2: Diagnostic performance of two commercial RDTs for SARS-Cov-2 antigen (part 2)** \| \| \| \| \| \| \| \| \| \| \| --- \| --- \| --- \| --- \| --- \| --- \| --- \| --- \| --- \| --- \| \| \|  \| **PPV (%)** \| \| **P*** \| **NPV (%)** \| \| **P*** \| **EFF (%)** \| \| **P*** \| \|  \| **Roche-RDT** \| **Abbott-RDT** \|  \| **Roche-RDT** \| **Abbott-RDT** \|  \| **Roche-RDT** \| **Abbott-RDT** \|  \| \| All probands \| 97·6 \| 99 \| 0·0717 \| 93·3 \| 92·8 \| <0·0001 \| 93·7 \| 93·3 \| 0·0037 \| \| Age > median \| 97·1 \| 98 \| 0·5867 \| 92·4 \| 92·2 \| 0·2975 \| 92·8 \| 92·7 \| 0·5433 \| \| Age ≤ median \| 98·1 \| 99·5 \| 0·7759 \| 94·3 \| 93·3 \| <0·0001 \| 94·6 \| 93·9 \| 0·0035 \| \| Women \| 98 \| 98·9 \| 0·5437 \| 93·2 \| 92·7 \| 0·0149 \| 93·6 \| 93·2 \| 0·0429 \| \| Men \| 97·2 \| 99 \| 0·1865 \| 93·4 \| 92·8 \| 0·0031 \| 93·8 \| 93·4 \| 0·0675 \| \| **Reason for testing** \|  \|  \|  \|  \|  \|  \|  \|  \|  \| \| Referral from physician \| 97 \| 99 \| 0·1377 \| 95·9 \| 95·7 \| 0·7907 \| 96 \| 96·2 \| 0·5533 \| \| Referral from Health Department (mostly contact persons of infected patients) \| 99 \| 98·8 \| 0·6819 \| 88·8 \| 87·8 \| 0·0003 \| 89·8 \| 88·8 \| 0·0003 \| \| Other \| 92·3 \| 95·7 \| 0·7045 \| 97·7 \| 97·6 \| 0·3329 \| 97·6 \| 97·5 \| 0·4843 \| \| **Comorbidities** \|  \|  \|  \|  \|  \|  \|  \|  \|  \| \| Any comorbidity \| 98 \| 98·9 \| 0·5627 \| 81·9 \| 81 \| 0·0025 \| 83·6 \| 82·7 \| 0·0107 \| \| No comorbidity \| 97·5 \| 98·7 \| 0·2111 \| 96·6 \| 96·2 \| 0·0071 \| 96·7 \| 96·4 \| 0·0589 \| \| Hypertension \| 97·4 \| 97·4 \| 1 \| 91·8 \| 91·8 \| 1 \| 92·3 \| 92·3 \| 1 \| \| Dyslipoproteinaemia \| 90·9 \| 95·2 \| 0·9748 \| 91·8 \| 91·8 \| 0·9748 \| 91·7 \| 92·1 \| 0·9748 \| \| Diabetes mellitus \| 83·3 \| 90·9 \| not defined \| 88·4 \| 88·5 \| 0·9906 \| 87·8 \| 88·8 \| 0·9908 \| \| COPD \| *** \| *** \| *** \| *** \| *** \| *** \| *** \| *** \| *** \| \| Ischaemic heart disease \| 85·7 \| 92·3 \| 0·9994 \| 95·5 \| 95·6 \| 0·9936 \| 93·1 \| 94·8 \| 0·9994 \| \| Previous COVID-19 \| 98·2 \| 97·9 \| 0·6527 \| 53·7 \| 52·1 \| 0·0071 \| 60·7 \| 58 \| 0·0071 \| \| **Clinical symptoms** \|  \|  \|  \|  \|  \|  \|  \|  \|  \| \| Any clinical symptoms \| 98·2 \| 98·8 \| 0·5469 \| 93·2 \| 92·9 \| 0·3247 \| 94 \| 93·9 \| 0·5727 \| \| No clinical symptoms \| 94·9 \| 98·2 \| 0·7659 \| 93·4 \| 92·7 \| <0·0001 \| 93·5 \| 92·8 \| 0·0011 \| \| Malaise \| 98·4 \| 99·2 \| 0·4423 \| 94·4 \| 94·3 \| 0·7251 \| 95·3 \| 95·3 \| 0·9938 \| \| Shortness of breath \| 98·2 \| 98·2 \| 1 \| 86·9 \| 86·9 \| 1 \| 88·7 \| 88·7 \| 1 \| \| Cough \| 99·5 \| 99·5 \| 0·6626 \| 91 \| 90·8 \| 0·4951 \| 92·9 \| 92·7 \| 0·4951 \| \| Fever \| 99 \| 99 \| 0·8711 \| 93·8 \| 92·8 \| 0·6775 \| 95·6 \| 95 \| 0·6775 \| \| Diarrhoea \| 95·5 \| 97·7 \| 0·9876 \| 91·7 \| 91·7 \| 0·9876 \| 92·2 \| 92·5 \| 0·9876 \| \| Musculoskeletal pain \| 98·9 \| 99·5 \| 0·999 \| 92·3 \| 92·3 \| 0·9926 \| 94·1 \| 94·2 \| 0·9946 \| \| Headache \| 98·1 \| 99 \| 0·4303 \| 95·1 \| 94·9 \| 0·7409 \| 95·6 \| 95·6 \| 0·9696 \| \| Nausea \| 95 \| 97·3 \| 0·7013 \| 94·5 \| 93·7 \| 0·3509 \| 94·6 \| 94·2 \| 0·4909 \| \| **Vaccination status** \|  \|  \|  \|  \|  \|  \|  \|  \|  \| \| Not vaccinated \| 98 \| 98·9 \| 0·2143 \| 92·9 \| 92·3 \| <0·0001 \| 93·4 \| 92·9 \| 0·0015 \| \| Vaccinated \| 91·7 \| 95·7 \| 0·9912 \| 97·8 \| 97·9 \| 0·987 \| 97·5 \| 97·7 \| 0·9888 \| \| Unknown \| *** \| *** \| *** \| *** \| *** \| *** \| *** \| *** \| *** \| \| **Vital signs** \|  \|  \|  \|  \|  \|  \|  \|  \|  \| \| SysBP>130 mmHg and/or SysBP>90 mmHg \| 97·1 \| 99·3 \| 0·4899 \| 92·9 \| 92·9 \| 0·4899 \| 93·3 \| 93·4 \| 0·4899 \| \| Other blood pressures \| 97·8 \| 98·4 \| 0·6227 \| 93·6 \| 92·7 \| <0·0001 \| 94 \| 93·2 \| 0·0011 \| \| Body temperature > 37°C \| 95·7 \| 95·7 \| 1 \| 93·9 \| 93·9 \| 1 \| 94·6 \| 94·6 \| 1 \| \| Body temperature ≤ 37°C \| 97·5 \| 98·9 \| 0·0715 \| 93·3 \| 92·8 \| <0·0001 \| 93·7 \| 93·3 \| 0·0065 \| \| Oxygen saturation > median \| 97·8 \| 97·8 \| 1 \| 93·8 \| 93·8 \| 1 \| 94·1 \| 94·1 \| 1 \| \| Oxygen saturation ≤ median \| 97·3 \| 98·8 \| 0·0589 \| 93·2 \| 92·6 \| 0·0003 \| 93·6 \| 93·2 \| 0·0047 \| \| **SARS-CoV-2 genotype (for Ct≤30)** \|  \|  \|  \|  \|  \|  \|  \|  \|  \| \| N501Y and delH69/V70 (Alpha variant, B.1.1.7) \| 100 \| 100 \| 1 \| 0 \| 0 \| 1 \| 77·1 \| 72·3 \| 0·0021 \| \| Variants of concern not found \|  \|  \| 1 \|  \|  \| 1 \| 87·7 \| 84 \| 0·0635 \| \| Other \| *** \| *** \| *** \| *** \| *** \| *** \| *** \| *** \| *** \| \|  \|  \|  \|  \|  \|  \|  \|  \|  \|  \| \| **** Roche versus Abbott (test on equality, based on 5000 bootstrap iterations) \| \| \| \| \|  \|  \|  \|  \|  \| \| **** No entry (division by zero, etc.). \| \| \| \| \|  \|  \|  \|  \|  \| \| **** Not defined due to low number. \| \| \| \| \|  \|  \|  \|  \|  \| \| **** Too low number of patients with negative/positive RDT. \| \| \| \| \|  \|  \|  \|  \|  \| \| PPV (predictive value of the positive tests): Proportion of true positive RDTs to all positive RDTs. \| \| \| \| \|  \|  \|  \|  \|  \| \| PNV (predictive value of the negative tests): Proportion of true negative RDTs to all negative RDTs. \| \| \| \| \|  \|  \| \| EFF (diagnostic efficiency): The ratio of correctly predicted and correctly excluded SARS-CoV-2. \| \| \| \| \|  \|  \|  \| |
| --- | --- | --- | --- | --- | --- | --- | --- | --- | --- | --- | --- | --- | --- | --- | --- | --- | --- | --- | --- | --- | --- | --- | --- | --- | --- | --- | --- | --- | --- | --- | --- | --- | --- | --- | --- | --- | --- | --- | --- | --- | --- | --- | --- | --- | --- | --- | --- | --- | --- | --- | --- | --- | --- | --- | --- | --- | --- | --- | --- | --- | --- | --- | --- | --- | --- | --- | --- | --- | --- | --- | --- | --- | --- | --- | --- | --- | --- | --- | --- | --- | --- | --- | --- | --- | --- | --- | --- | --- | --- | --- | --- | --- | --- | --- | --- | --- | --- | --- | --- | --- | --- | --- | --- | --- | --- | --- | --- | --- | --- | --- | --- | --- | --- | --- | --- | --- | --- | --- | --- | --- | --- | --- | --- | --- | --- | --- | --- | --- | --- | --- | --- | --- | --- | --- | --- | --- | --- | --- | --- | --- | --- | --- | --- | --- | --- | --- | --- | --- | --- | --- | --- | --- | --- | --- | --- | --- | --- | --- | --- | --- | --- | --- | --- | --- | --- | --- | --- | --- | --- | --- | --- | --- | --- | --- | --- | --- | --- | --- | --- | --- | --- | --- | --- | --- | --- | --- | --- | --- | --- | --- | --- | --- | --- | --- | --- | --- | --- | --- | --- | --- | --- | --- | --- | --- | --- | --- | --- | --- | --- | --- | --- | --- | --- | --- | --- | --- | --- | --- | --- | --- | --- | --- | --- | --- | --- | --- | --- | --- | --- | --- | --- | --- | --- | --- | --- | --- | --- | --- | --- | --- | --- | --- | --- | --- | --- | --- | --- | --- | --- | --- | --- | --- | --- | --- | --- | --- | --- | --- | --- | --- | --- | --- | --- | --- | --- | --- | --- | --- | --- | --- | --- | --- | --- | --- | --- | --- | --- | --- | --- | --- | --- | --- | --- | --- | --- | --- | --- | --- | --- | --- | --- | --- | --- | --- | --- | --- | --- | --- | --- | --- | --- | --- | --- | --- | --- | --- | --- | --- | --- | --- | --- | --- | --- | --- | --- | --- | --- | --- | --- | --- | --- | --- | --- | --- | --- | --- | --- | --- | --- | --- | --- | --- | --- | --- | --- | --- | --- | --- | --- | --- | --- | --- | --- | --- | --- | --- | --- | --- | --- | --- | --- | --- | --- | --- | --- | --- | --- | --- | --- | --- | --- | --- | --- | --- | --- | --- | --- | --- | --- | --- | --- | --- | --- | --- | --- | --- | --- | --- | --- | --- | --- | --- | --- | --- | --- | --- | --- | --- | --- | --- | --- | --- | --- | --- | --- | --- | --- | --- | --- | --- | --- | --- | --- | --- | --- | --- | --- | --- | --- | --- | --- | --- | --- | --- | --- | --- | --- | --- | --- | --- | --- | --- | --- | --- | --- | --- | --- | --- | --- | --- | --- | --- | --- | --- | --- | --- | --- | --- | --- | --- | --- | --- | --- | --- | --- | --- | --- | --- | --- | --- | --- | --- | --- | --- | --- | --- | --- | --- | --- | --- | --- | --- | --- | --- | --- | --- | --- | --- | --- | --- | --- | --- | --- | --- | --- | --- | --- | --- | --- | --- | --- | --- | --- | --- | --- | --- | --- | --- | --- | --- | --- | --- | --- | --- | --- | --- | --- | --- | --- | --- | --- | --- | --- | --- | --- | --- | --- | --- | --- | --- | --- | --- | --- | --- | --- | --- | --- | --- | --- | --- | --- | --- | --- | --- | --- | --- | --- | --- | --- | --- | --- | --- | --- | --- | --- | --- | --- | --- | --- | --- | --- | --- | --- | --- | --- | --- | --- | --- | --- | --- | --- | --- | --- | --- | --- | --- | --- | --- | --- | --- | --- | --- | --- | --- | --- | --- | --- | --- | --- | --- | --- | --- | --- | --- | --- | --- | --- | --- | --- | --- | --- | --- | --- | --- | --- | --- | --- | --- | --- | --- | --- | --- | --- | --- | --- | --- | --- | --- | --- | --- | --- | --- | --- | --- | --- | --- | --- | --- | --- | --- | --- | --- | --- | --- | --- | --- | --- | --- | --- | --- | --- | --- | --- | --- | --- | --- | --- | --- | --- | --- | --- | --- | --- | --- | --- | --- | --- | --- | --- | --- | --- | --- | --- | --- | --- | --- | --- | --- | --- | --- | --- | --- | --- | --- | --- | --- | --- | --- | --- | --- | --- | --- | --- | --- | --- | --- | --- | --- | --- | --- | --- | --- | --- | --- | --- | --- | --- | --- | --- | --- | --- | --- | --- | --- | --- | --- | --- | --- | --- | --- | --- | --- | --- | --- | --- | --- | --- | --- | --- | --- | --- | --- | --- | --- | --- | --- | --- | --- | --- | --- | --- | --- | --- | --- | --- | --- | --- | --- | --- | --- | --- | --- | --- | --- | --- | --- | --- | --- | --- | --- | --- | --- | --- | --- | --- | --- | --- | --- | --- | --- | --- | --- | --- | --- | --- | --- | --- | --- | --- | --- | --- | --- | --- | --- | --- | --- | --- | --- | --- | --- | --- | --- | --- | --- | --- | --- | --- | --- | --- | --- | --- | --- | --- | --- | --- | --- | --- | --- | --- | --- | --- | --- | --- | --- | --- | --- | --- | --- | --- | --- | --- | --- | --- | --- | --- | --- | --- | --- | --- | --- | --- | --- | --- | --- | --- | --- | --- | --- | --- | --- | --- | --- | --- | --- | --- | --- | --- | --- | --- | --- | --- | --- | --- | --- | --- | --- | --- | --- | --- | --- | --- | --- | --- | --- | --- | --- | --- | --- | --- | --- | --- | --- | --- | --- | --- | --- | --- | --- | --- | --- | --- | --- | --- | --- | --- | --- | --- | --- | --- | --- | --- | --- | --- | --- | --- | --- | --- | --- | --- | --- | --- | --- | --- | --- | --- | --- | --- | --- | --- | --- | --- | --- | --- | --- | --- | --- | --- | --- | --- | --- | --- | --- | --- | --- | --- | --- | --- | --- | --- | --- | --- | --- | --- | --- | --- | --- | --- | --- | --- | --- | --- | --- | --- | --- | --- | --- | --- | --- | --- | --- | --- | --- | --- | --- | --- | --- | --- | --- | --- | --- | --- | --- | --- | --- | --- | --- | --- | --- | --- | --- | --- | --- | --- | --- | --- | --- | --- | --- | --- | --- | --- | --- | --- | --- | --- | --- | --- | --- | --- | --- | --- | --- | --- | --- | --- | --- | --- | --- | --- | --- | --- | --- | --- | --- | --- | --- | --- | --- | --- | --- | --- | --- | --- | --- | --- | --- | --- | --- | --- | --- | --- | --- | --- | --- | --- | --- | --- | --- | --- | --- | --- | --- | --- | --- | --- | --- | --- | --- | --- | --- | --- | --- | --- | --- | --- | --- | --- | --- | --- | --- | --- | --- | --- | --- | --- | --- | --- | --- | --- | --- | --- |
|  |

**Supplementary Figure 1. Choropleth map of GISD (German Index of Socio-Economic Deprivation) strata and residence areas of study particiants**


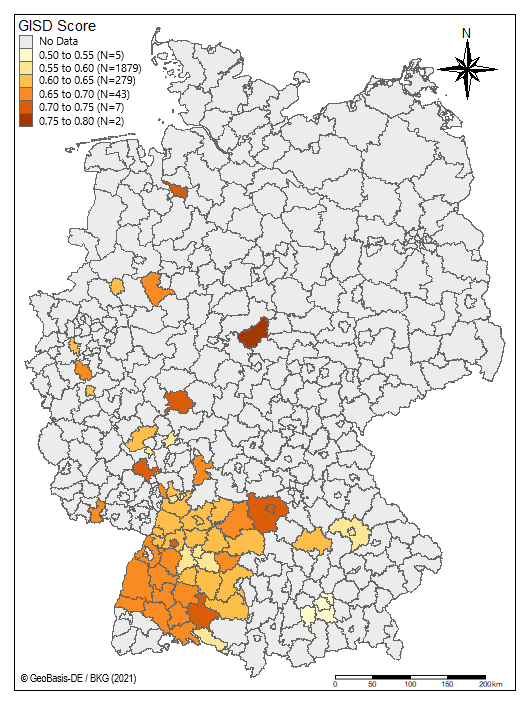

Supplement: Supplementary file 1 [file Data_Sheet_1.docx]
